# Supplementary material for: Patterns of Intron Gain and Loss in Fungi
Source: PLoS Biol. 2004 Nov 30;2(12):e422. doi: 10.1371/journal.pbio.0020422 (PMC532390; doi:10.1371/journal.pbio.0020422)
Supplement: Table S1 — Also available at http://genes.mit.edu/NielsenEtAl/. (4.3 MB ZIP). [file pbio.0020422.st001.zip › NielsenEtAl/html/983.html]

AN5815.1.NCU00108.1.MG00479.1.FG06959.1


```
 CLUSTAL W (1.82) Multiple Sequence Alignments - Introns Inserted


Sequence 1: MG00479.1	397 aa
Sequence 2: FG06959.1	393 aa
Sequence 3: NCU00108.1	411 aa
Sequence 4: AN5815.1	390 aa
Alignment Length: 421 aa
Number Identitical Residues: 212 aa
Alignment Score (without introns) 9956


MG00479.1 	MATKAMQLEECFEHLNVNDEND---HGDRLVQ--~---KSK0ISMS----TAQISHGSSR
NCU00108.1	MAGQ--PMESRFGRMSIHDENNDPSDGARLQQKA~RELKSK0IAQR----KNPPIISSEP
FG06959.1 	MATR--TIEARFERMSVNDENDPIGDGSKLYS--~---KSK0VQTI----SSTSSHGANR
AN5815.1  	MATK--TLEARFEHLSVKDDSGNGNKGTSYPKHK0SSLSTA~VSLSGLGVAGQLTSGANR
          	** :   :*  * ::.::*:..  ..*    .    . .:  :   . .       .:. 

MG00479.1 	PNLFK------VALQSQSANTVTSVTLPSQAAQRKMAPPPKSREPPSPSKHKKDSVSSSV
NCU00108.1	PSPRKPQRTVPVATKSSTIVSSAAHKSTTYAGIRRVDDDD----HDELSQPMASASVSST
FG06959.1 	PNLFK------VALQSQSSNANAAVMLPSQAAQRKVNNPP-----PSPTRKALPSSSRTS
AN5815.1  	SNLLK------LALQNTNDNKVNAMNANASQSPGKGTQNP--------------ASLRNL
          	..  *      :* :. .     :    :  .  :                   :   . 

MG00479.1 	DELANEEVEASAPR---VPKEFHLGMFEIGRPLGKGKFGRVYLARERTTGFICALKVLYK
NCU00108.1	GTSTSESSSSAGYEQSISPKQFHLGMFEIGRPLGKGKFGRVYLARERSSGFICALKVLYK
FG06959.1 	DEVSEDRKSVTLPEQMSVPKQFHLGMFEIGRPLGKGKFGRVYLARERTTGFICALKVLHK
AN5815.1  	DENGEYEQP--------APRKLHLGMFEIGKPLGKGKFGRVYLAKERSSGFVCALKVLHK
          	.   .             *:::********:*************:**::**:******:*

MG00479.1 	KELESGS-VEKQVRREIEIQTNLCHPNILKMYGHFHDNKRIFLILEFAGKGELYKHLRKE
NCU00108.1	SELQHGTGVEKQVRREIEIQSNLRHPNILKLYGHFHDSKRIFLILEYAGKGELYKHLRKE
FG06959.1 	NELQAGR-VEKQVRREIEIQSNLRHPNILQLYGHFHDSKRVFLILEFAGKGELYKHLRKE
AN5815.1  	SELQQGG-VQKQVRREIEIQSNLRHPNVLRLFGHFQDSKRIFLILEFAGRGELYKHLRKE
          	.**: *  *:**********:** ***:*:::***:*.**:*****:**:**********

MG00479.1 	NRFPEWKAAQYVAQMASALKYLHRKHVIHRDIKPENILVGLHGELKISDFGWSVHAPNNR
NCU00108.1	NRFPEWKAAQYVAQMASALKYLHRKHVIHRDIKPENILVGIHGEIKISDFGWSVHAPNNR
FG06959.1 	NRFPEWKAAQYIAQMASALRYLHRKHVIHRDIKPENILVGIHGEIKISDFGWSVHAPNNR
AN5815.1  	HRFPEWKAAHYIAQMASALKYLHKKHVMHRDIKPENILVGIHGEIKISDFGWSVHAPNNR
          	:********:*:*******:***:***:************:***:***************

MG00479.1 	RKTLCGTLDYLPPEMIKSGNKDNTYDEKVDLWSLGVLTYEFVVGEAPFEDTPVMTQRRIA
NCU00108.1	RQTLCGTLDYLPPEMIRSGSKDNWYNEKVDLWSLGVLTYEFLVGEAPFEDTPIMTQKRIA
FG06959.1 	RKTMCGTLDYLPPEMIKPGTSDNFYNEKVDLWSLGVLTYEFLVGEAPFEDTPVMTQRRIA
AN5815.1  	RQTMCGTLDYLPPEMLTSNPQGNFYNEKVDLWSLGVLTYEFLVGEAPFEDTPVMTQRRIQ
          	*:*:***********: .. ..* *:***************:**********:***:** 

MG00479.1 	RADMTIPSFVSPECRDLIKK0LLVLDPVKRLSLDAVQKHPWIIKHCVKGERATNREKG~R
NCU00108.1	RADMTIPEWVSKEAKDLIKK0LLVLDPEKRLPLEEVENHPWILKHCVKGERAANREKM~A
FG06959.1 	RADMSIPTWVSPEATDLIKK0LLVLDPEKRIPLEQIQQHPWIIKHCVKGERASNREKG~L
AN5815.1  	RGDMQVPSFVSPEAKDLIKR0LLVLDPEKRISLDEIQRHPWILKHCVKDDRTIKRGSA1P
          	*.** :* :** *. ****: ****** **:.*: ::.****:*****.:*: :* .   

MG00479.1 	GSD--
NCU00108.1	SKMGN
FG06959.1 	-----
AN5815.1  	HAANE
          	   ..
```
